# Infant Symptoms and Modification of Food

**Published:** 1900-04-28

**Authors:** Eric Pritchard


### Hospital Clinics and Medical Progress.

### INFANT SYMPTOMS AND MODIFICATION OF FOOD.

By Eric Pritchard, M.A., M.D., Oxon.

As far as possible the food of an artificially-fed infant should conform to the standard of human milk; that is to say, as regards the proportion of its three main constituents—namely, fat, proteid, and sugar. As regards quantity it should be regulated according to the physiological needs of each particular case. In the great majority of cases if these conditions are fulfilled the child's condition will be satisfactory; but in certain cases, owing to idiosyncracies or other unexplained causes, the infant's progress may be unsatisfactory, and in such cases changes or modification in the food may be required. Perhaps all that may be necessary may be some slight alteration in the proportions of the fat, proteids, or sugar contained in its mixture of modified cow's milk; or it may be found that no form of cow's milk, modified or otherwise, is suitable for the particular case. In such an event an alternative variety of food must be tried; but whatever form the change take it is essential that the food contain representatives of the three chief elements of milk, and that they be present, at any rate in the first place, in the physiological proportions. Starting with this as a basis, modifications may subsequently be made with definite ends in view, and with full appreciation of the indications.

The accompanying table contains the directions for preparing six alternative varieties of foods suitable for infants, and each contains fat, proteid, and sugar in the physiological proportions of human milk. Recourse should not, however, be had to any one of these alternative diets until it is definitely known that the infant cannot digest and assimilate any variety of modified cow's milk, and that no such extraneous complication as teething or vaccination is interfering with normal nutrition:—

Table of Six Mixtures suitable for delicate and sick infants. They each contain fat, proteids, and carbo-hydrates in the same proportion as they exist in average human milk, *i.e.*, fat, 4 per cent.; proteid, 1·5 per cent.; sugar, 6·5 per cent.

No. 1. Peptonised Milk.—Milk, 4 oz.; cream, 3 oz. 4 dr.; milk sugar, 1 oz. 2 dr. (by measure, not weight). Water to make one pint. To be peptonised for half an hour with Liquor Pancreaticus (Benger), stirring constantly.

No. 2. Fresh Meat Juice.—Meat juice, 2 oz.; cream, 5 oz.; milk sugar, ½ lb. (by measure, not weight). Water to make one pint. To make the meat juice: Take 1 oz. 3 dr. minced rump steak, well mixed with 2 oz. of water; let it stand for half hour, and then express juice. Meat juice made in this way contains 5 per cent. proteid.

No. 3. Condensed Milk.—Condensed milk, 2 oz.; cream, 3 oz. 4 dr. Water to make one pint. (According to Meigs, a good variety of condensed milk contains: Fat, 10 per cent.; casein, 9 per cent.; sugar, 50 per cent.)

No. 4. Horlick's Malted Milk.—Horlick's malted milk, 1 oz.; cream, 4 oz. 4 dr.; milk sugar, 5 dr. Water to make one pint.

No. 5. White of Egg Mixture.—White of egg, 1 oz.; cream, 5 oz.; milk sugar, 1 oz. 3 dr. (by measure, not weight). Water to one pint. Measure 1 oz. white of egg, cut in various directions with clean scissors, shake well with 13 oz. of cold water, strain through muslin, and add cream and sugar.

No. 6. Asses' Milk.—Asses' milk, 17 oz.; cream (cow's), 3 oz. 4 dr.

| Age of Infant. | 3–14 days. | 14–28 days. | 2 mths. | 3 mths. | 4–6 mths. | 6–8 mths. | 8 mths. |
|---|---|---|---|---|---|---|---|
| Quantity of any of above mixtures for 24 hours not to exceed... | 12 oz. | 18 oz. | 24 oz. | 30 oz. | 36 oz. | 42 oz. | 48 oz. |

When the progress of an infant is apparently unsatisfactory we are most likely to gain definite information as to the cause by an examination of the motions, the character of the weight chart,* and by enquiring whether vomiting is a symptom. An infant should show during the first six months of life a weekly increment in weight of from five to eight ounces. Continued increments in weight above or below these figures are not auspicious: if above eight ounces the infant is probably being overfed; if below, either too little food is being supplied, or what is more probable, too little is being digested and assimilated—an examination of the motions will generally decide which is the case. If the infant is receiving too little food the motions will be small, constipated, dry, and well digested, and there may be an elevation of temperature; if the infant cannot digest and assimilate the food the motions will be copious, and contain the debris of the element or elements of the food which is disagreeing. The most frequent element of the food which defies digestion is the casein or milk curd. If this be the case small particles of white coagulum will be present in the motions. It is possible sometimes to confound shreds of mucous or particles of fat for pieces of undigested clot, and the mistake is especially liable to occur if the motions are examined some time after they have passed and have become cold. Simple microscopical or chemical tests will at once settle the question in cases of doubt. If genuine particles of casein are found in the stools, it is necessary to reduce the quantity of proteid in the food or supply it in some other form; the first can be effected by reducing the quantity of milk, and the second by peptonising it or giving one of the alternative food mixtures. It is probably the better plan to start by reducing the proteid half per cent. at a time, and if this is not found effectual after a fair trial to proceed to one of the alternative diets, reverting as soon as possible to modified milk of the correct standard.

Indications may be found in the motions that the infant is receiving either too much or too little of the fatty element in its food. Either of these errors may affect the general nutrition. If there is a deficiency of fat the motions will be dry and brittle, with constipation and straining. If excess of fat there may be diarrhœa, vomiting of sour ingesta, and the motion will be greasy and rancid in odour. Either of these mistakes are easily corrected by increasing or decreasing the percentage of cream in the food.

Excess or deficiency of the carbo-hydrate element of

* An infant's weight should be recorded weekly; and for keeping the record "The Infants' Weight Chart," published by Reynolds and Branson, Briggate, Leeds, will be found most useful.

the food may have a marked influence on the condition of health of an infant. Excess is generally evidenced by the too rapid increase in weight of the infant; that is to say up to a certain point, if the excess exceeds a definite limit diarrhœa will occur, the stools will be frothy from admixture with gases of decomposition, they will smell extremely sour, and prove strongly acid to litmus paper. Deficiency of carbohydrates in the food can only be proved by the exclusion of other possible causes of failure of nutrition. It may be suspected when the motions are normal, and the infant is satisfactory in other respects, except that its weekly increment in weight is below the normal. Excess or deficiency in this element of the food can be accurately and easily rectified by decreasing or increasing the amount of milk sugar in the mixture. To modify the proportions of proteid, fat, and sugar in exact percentages, reference must be made to a special table for preparing milk mixtures.

There are certain common conditions of the bowel which are not dependent on errors of diet, and which cannot be satisfactorily treated by dietetic means. The original exciting cause may perhaps have been due to some temporary mistake, but the permanent cause which keeps up the condition is some pathological state of the bowel. The diarrhœa of chronic or subacute colitis is a case in point; it is generally set up by fermentative processes in the bowel, which lead to catarrh of the mucous membrane, but cold or chill may equally well bring about the same condition. No modification of the diet, provided it is physiologically sound, is likely to prove of any immediate advantage. The diarrhœa of colitis may be distinguished from forms of diarrhœa due to dietetic errors by the following characters: The motions appear well digested, rather liquid, but normal in colour; they are, however, accompanied or followed by the discharge of a large quantity of mucous. This mucous, unlike that of catarrh of higher portions of the bowel, is not intimately mixed with the motions; the mucous is separate, perhaps coats, or follows, the ejecta; it may be blood-stained, and there may be prolapse of the bowel. Then with regard to constipation; this frequently occurs in a form which is not amenable to treatment by changes in the diet—in such cases it is generally due to inertia of the bowel—it generally succeeds the diarrhœa of colitis, when that condition is cured; it sometimes ensues on the use of enemas or glycerine suppositories, which dilate the lower portion of the bowel or dull its normal sensitiveness. Constipation from inertia of the bowel is very persistent; the bowels may be only moved every second, third, or fourth day. In consistency the motions are quite normal and well-digested, but very copious. This condition is better treated by the medical expedients of rhubarb or cascara than by tampering with a diet which is in no way at fault.

Vomiting as an independent symptom may usually be regarded as a discreditable reflection on the method of feeding. It is nearly always preventable, and should be prevented; the most prolific source is undoubtedly excess in the actual bulk of food given at each feeding. The habit of vomiting is easily acquired during the early days of life, when the stomach is hypersensitive and intolerant of liberties, and it is at this time that the stomach, relatively to its capacity, is more seriously overtaxed than at any subsequent period. This mechanical interference with the function of the stomach should be guarded against at all cost, as such habits of vomiting are not easily cured after they are once fairly established. Vomiting sometimes occurs from intolerance of particular forms of food, excess of fat, or the bulky coagulum of cow's milk being particularly liable to induce the habit. Limiting the cream will correct the former error, and the latter, perhaps, may be avoided by diluting the milk with barley water instead of plain water. Vomiting occurring as an associated symptom with general gastric intestinal catarrh is a more serious complication, and must be treated on special lines.

Rickets in the majority of cases is unquestionably a food disease, although the condition is intensified and aggravated by bad hygienic surroundings. It almost invariably follows any failure of the normal digestive processes, and, seeing how readily these are upset by injudicious feeding, it is not to be wondered at that food plays such an important rôle in the production of the disease. Overfeeding, whether as regards all the elements of the food or some one particular element, is perhaps the most common cause of all. Boiling or sterilisation of the milk is by some regarded as a factor in the production of this disease; if it has any influence at all it must be a slight one, considering the number of children that are brought up on milk which has been submitted to one or other of these processes and yet never show a symptom of the disease. However, when symptoms of the disease have declared themselves, it is beyond question that children improve more rapidly on fresh food that has not been subjected to high temperatures or prolonged boiling, and, in view of this, such a food mixture as No. 2, which contains raw meat juice, is particularly useful.

In supplying an infant with any mixture containing milk or cream, it must be remembered that artificial preservatives are sometimes added to keep them from turning sour. Such preservatives generally take the form of boracic or formic acid, or some of their numerous derivatives. The presence of such extraneous substances in the food cannot but be prejudicial to the health of a delicate child, and hence, should all obvious causes of malnutrition be absent, suspicion may legitimostly be aroused as to contamination with some such body.

